# Awareness of Medication-Related Osteonecrosis of the Jaws Amongst Patients on Antiresorptive and Antiangiogenic Medications

**DOI:** 10.7759/cureus.52896

**Published:** 2024-01-25

**Authors:** Ahmad S Assari, Yosef Alanazi, Elaf Mubarak Algharbi, Abdulmajeed Abuhabsha, Basel Alshammry, Ali Alzahrani, Abdulrahaman Alduhaim, Reem Abuhaimed

**Affiliations:** 1 Department of Oral Maxillofacial Surgery and Diagnostic Sciences, Riyadh Elm University, Riyadh, SAU; 2 Department of Oral and Maxillofacial Surgery, University of Hail, Hail, SAU; 3 Department of Preventive Dental Science, College of Dentistry, King Saud bin Abdulaziz University for Health Sciences, Riyadh, SAU; 4 Department of Preventive Dental Science, King Abdullah International Medical Research Center, Ministry of National Guard Health Affairs, Riyadh, SAU; 5 Department of Oral and Maxillofacial Surgery, King Saud University, Riyadh, SAU; 6 Department of Oral and Maxillofacial Surgery, Ministry of Health, Riyadh, SAU; 7 Department of Dentistry, King Saud University, Riyadh, SAU

**Keywords:** patient education, dental care for medicatpatient educationion users, bisphosphonates, oral health risks, patient awareness, mronj, jaws osteonecrosis, medication-related osteonecrosis

## Abstract

Background

Medication-related osteonecrosis of the jaws (MRONJ) is a rare but severe condition that has garnered increasing attention in recent years. It primarily affects individuals undergoing treatment with antiresorptive and antiangiogenic medications, such as bisphosphonates and denosumab, commonly prescribed for osteoporosis and cancer-related bone metastases. Therefore, the present study aimed to assess awareness and understanding of MRONJ among patients receiving antiresorptive and antiangiogenic medications.

Methods

A cross-sectional survey was conducted among 110 patients receiving antiresorptive and antiangiogenic medications in a clinical setting. Participants were given a structured questionnaire to assess their awareness of MRONJ. The questionnaire covered aspects such as MRONJ, bisphosphonate usage, and awareness of the condition's potential complications. Demographic information was also collected. Chi-square and Fisher's tests were performed using SPSS statistical software.

Results

In terms of gender distribution, 63.6% of the participants were female. Concerning age distribution, the majority (43.6%) fell within the 21 to 40 age group, whereas only 5.5% were aged over 60. Regarding educational attainment, a substantial majority (58.2%) of the participants held a bachelor's degree. The study findings reveal that a considerable proportion (35.5%) of participants possess awareness regarding jaw osteonecrosis, and this association is statistically significant (p=0.002). A substantial number of participants administered the medication orally (30.9%), while others utilized various administration routes, including injection (IV and others) (40%), and this difference was also statistically significant (p=0.001). Most participants took bisphosphonates for osteoporosis (41.8%) or cancer (13.6%), both statistically significant (p<0.01). Gender had no significant impact (p>0.01), but age showed potential associations (p=0.07 for awareness, p=0.003 for medication use). Educational backgrounds had no significant link, except for bisphosphonate usage (p<0.01) and side effects reporting (p<0.01).

Conclusion

Notably, a small percentage of participants demonstrated awareness of this condition, indicating a need for continued education and awareness campaigns. Further research and interventions may be warranted to address the specific needs of different age groups and educational backgrounds in promoting safe and effective medication management.

## Introduction

Osteoporosis is the most common chronic metabolic skeletal disorder, affecting bone by decreasing bone strength and mass, which leads to a higher incidence of bone fractures [1]. It is a silent disorder with no symptoms unless fractures occur; older individuals and females are common risk factors for developing osteoporosis [2]. This widespread disease can affect people of different ages and ethnicities, but it primarily affects the older age group. Osteoporosis results from more bone resorption than bone formation due to aging, affecting patients' mobility and increasing the possibility of fragile fractures. The global incidence of osteoporosis in men is 11.7%, with the highest rates documented in Africa, reaching 39.5% [3]. In Saudi Arabia, it is far more common than in the rest of the world, with a prevalence of 63.6% in men and 58.4% in postmenopausal women. Patients with osteoporosis have shown significant changes in their lifestyle since being diagnosed with the disease. These changes can inhibit the patient’s daily activities or lead to undesirable clinical outcomes [4-5]. Osteoporotic patients have shown significant changes in their lifestyle since they were diagnosed with this disease. These changes could inhibit the patient’s daily activities or cause undesirable clinical results [5]. Antiresorptive treatments are employed to enhance bone density in individuals diagnosed with osteoporosis [6]. These therapies encompass five primary categories of medications: bisphosphonates, estrogens, selective estrogen receptor modulators (SERMs), calcitonin, and monoclonal antibodies like denosumab [6]. They are used mainly in patients with osteoporosis and primary and secondary skeletal malignancies to decrease the incidence of fractures, pain, and metastatic spread. The most commonly used antiresorptive drug is bisphosphonates, including the current nitrogen-containing bisphosphonates (alendronate, pamidronate, risedronate, ibandronate, and zoledronate) [7]. Bisphosphonates work at a cellular level, targeting osteoclasts and disrupting their functions [8]. Intravenous bisphosphonates, such as pamidronic acid and zoledronic acid, gained FDA approval in 1996 and 2002, respectively [9,10]. The use of bisphosphonates in cases of osteoporosis has shown a positive effect in inhibiting bone fractures [11]. However, multiple undesirable side effects have been reported in various systems, necessitating suitable administration methods [12].

Medication-related osteonecrosis of the jaw (MRONJ) is a severe complication of antiresorptive and antiangiogenic agents, involving bone destruction in facial areas. The diagnosis of MRONJ relies on the history of clinical and radiological characteristics of bone destruction [13,14]. A new type of osteonecrosis, described in 2003 as BRONJ, refers to necrosis of the jaw bone and face region associated with the use of bisphosphonate drugs as antiresorptive or antiangiogenic medication for more than two months [15]. Bisphosphonates, antiresorptive, and antiangiogenic drugs are all well-known causative agents for MRONJ. However, a specific pathogen has not been reported. Multiple hypotheses have been proposed, including those related to oxidative stress therapy [16].

Awareness of MRONJ among patients on antiresorptive and antiangiogenic medications is paramount due to its potential for severe and debilitating consequences. These medications, commonly prescribed for conditions such as osteoporosis and cancer, can disrupt the delicate balance of bone remodeling and angiogenesis, predisposing patients to the development of MRONJ. This rare but devastating condition is characterized by bone tissue death in the jaws [8]. Early recognition and understanding of MRONJ risks empower patients to proactively engage in their healthcare decisions. It prompts healthcare providers to closely monitor and manage their oral health, and guides clinicians in optimizing treatment strategies, ultimately enhancing patient outcomes and quality of life while minimizing the risks associated with these essential medications. Unfortunately, awareness among patients regarding MRONJ has been estimated to be quite low in previous studies, ranging from as low as 9.2% to a high of 33.82% [5,17-19]. Therefore, the present study assessed awareness and understanding of MRONJ among patients receiving antiresorptive and antiangiogenic medications.

## Materials and methods

In this cross-sectional descriptive study, validated and standardized hard copies of self-administered questionnaire forms were utilized to gather data from Saudi Arabian individuals aged over 18 years who were willing to participate in the study and had given their consent. The participants visiting the Department of Oral and Maxillofacial Surgery and the Department of Diagnostic Sciences at Riyadh Elm University, as inpatients or outpatients from January 2022 to March 2022, were provided with a self-administered questionnaire and a cover letter elucidating the study's objectives, adapted from previous studies [5,17-20]. The questionnaire comprised two sections: the first part gathered information on demographic variables such as age, gender, and education, while the second part measured awareness levels concerning MRONJ. The second part had eight questions, among which two offered only two options, ‘Yes’ or ‘No’; two presented three options, ‘Yes’, ‘No’, or ‘I don’t know’; and the other four had multiple different options. Of these, two questions allowed multiple responses, while the remaining four required choosing only one option. There were three questions related to awareness of the side effects of the antiresorptive and antiangiogenic medications they were taking. Respondents were not obligated to disclose their names on the questionnaire to ensure complete confidentiality. The convenience sampling method was used, and the study encompassed 110 participants.

Ethical considerations

The study was approved by the Institutional Review Board of Riyadh Elm University with the approval number SRP/2022/120/765/738 and was conducted in accordance with the principles of the Declaration of Helsinki.

Data analysis

Descriptive statistics were used to calculate the frequency distributions and percentages of the categorical variables. Chi-square and Fisher's exact tests were utilized to assess the association between the characteristics of the study participants and their knowledge, as well as their responses to bisphosphonate-related items. A significance level of less than 0.05 was adopted for all statistical tests. The data were analyzed using the SPSS version 21 (IBM Corp., Armonk, NY, USA).

## Results

The survey collected responses from 110 participants. In terms of age distribution, 20% of respondents were under 20 years old, 43.6% were between 21 and 40, 30% were between 41 and 60, and a smaller percentage, 5.5%, were over 60. The gender distribution showed that 36.4% identified as male, while 63.6% identified as female. Regarding education, the majority of participants held a bachelor's degree (58.2%), followed by high school education (25.5%), postgraduate degrees (7.3%), and the smallest percentage having completed elementary education (9.1%) (Table 1). These demographics provide valuable insights into the composition of the survey respondents, aiding in understanding how different groups perceive the subject matter.

**Table 1:** Demographic characteristics of respondents.

Variables	Frequency	Percentage
Age (years)		
<20	22	20
21-40	48	43.6
41-60	33	30
>60	6	5.5
Gender		
Male	40	36.4
Female	70	63.6
Education		
Elementary	10	9.1
High school	28	25.5
Bachelors	64	58.2
Postgraduate (Higher education)	8	7.3

The study results show that a significant proportion (35.5%) of participants are aware of jaw osteonecrosis, with a statistically significant association (p=0.002). Regarding bisphosphonate usage, the majority of participants (47.3%) had not taken them. Those who had were distributed among different categories: 'I used in the past' (20%), 'Yes, taking now' (9.1%), and 'No, but maybe I will' (3.6%), with a statistical significant association (p=0.01). Many participants took the medicine orally (30.9%), while others used different routes, including injection, IV, and others (40%), with a significant association (p=0.001). When asked about being transitioned during initiation, a small percentage confirmed being transitioned (6.4%), a significant portion was uncertain (44.5%), and 20% were not being transitioned, with a significant association (p=0.001). The main reasons for taking bisphosphonates were osteoporosis (41.8%) and cancer (13.6%), with p<0.01. About 23.6% of participants did receive, and 22.7% didn’t receive information about side effects, with the responses showing non-significant associations (p=0.98). The primary source of this information was doctors (35.5%, p=0.001). The reported side effects varied, with 'None' (17.3%), 'Pain' (12.7%), and 'I don’t know' (30.9%) being the most prominent responses, with a significant p-value (Table 2).

**Table 2:** Association between responses. Statistical significance at p≤0.05.

Question No.	Parameters	Response	Number	Percentage	P-value
1.	Do you know what osteonecrosis of the jaw is?	Yes	39	35.5	0.002
		No	71	64.5	
2.	Have you ever taken bisphosphonates or any of the antiresorptive and antiangiogenic medications?	No, I don’t think I will	52	47.3	0.01
		No, maybe I will	4	3.6	
		I used in the past	22	20	
		Yes, taking now	10	9.1	
		I don’t know	22	20	
3.	What was the route of administration?	Oral	34	30.9	0.001
		Injection	12	11.1	
		IV	4	3.7	
		None	0	0	
		Others	44	40	
4.	Were you transitioned to a different medication when you first began treatment?	Yes	7	6.4	0.001
		No	22	20	
		I don’t know	49	44.5	
5.	For which condition have you taken the medicine?	Cancer	15	13.6	0.001
		Osteoporosis	46	41.8	
		Others	8	7.3	
6.	Did you receive information about the side effects?	Yes	26	23.6	0.98
		No	25	22.7	
		I don’t know	26	23.6	
7.	Source of side effect information	Doctor	39	35.5	0.001
		Assistant	0	0	
		Dentist	13	11.8	
		Nurse	2	1.8	
		Pharmacist	4	3.6	
		Others	13	11.8	
8.	What are the side effects?	None	19	17.3	0.001
		Pain	14	12.7	
		Infection	3	2.7	
		Swelling	2	1.8	
		Exposed bone	5	4.5	
		I don’t know	43	30.9	
		Others	1	0.9	

The results of the demographic analysis revealed several noteworthy findings. In terms of age groups, the p-values for the questions related to awareness of osteonecrosis of the jaw and the usage of medication taken are 0.07 and 0.003, respectively, suggesting potential associations with age. However, the p-values for questions regarding the condition used, transitioning during treatment initiation, receiving information about side effects, and knowing the source of side effect information did not show significant differences across age groups. Similarly, the gender-wise analysis displayed non-significant variations for all questions (p>0.01). Additionally, the educational background of respondents had a noteworthy connection to their responses, showing a non-significant association (p>0.01) except for bisphosphonate usage (p<0.001) and side effects (p<0.01) (Table 3). Overall, these results suggest that demographics did not significantly influence respondents' awareness, treatment practices, and information sources regarding osteonecrosis of the jaw and bisphosphonate medications.

**Table 3:** Association of demographic characteristics with awareness questions asked. Statistical significance at p≤0.05.

Demographics	Questions	P-value	Fisher’s test
Age (years)			
<20, 21-40, 41-60, >60	Do you know what osteonecrosis of the jaw is?	0.07	0.06
	Have you ever taken any of the bisphosphonates or any of the antiresorptive and antiangiogenic medications?	0.003	0.004
	What is the route of administration?	0.12	0.03
	When you first began taking the drug, were you transitioned from another drug?	0.25	0.17
	For which condition do you take the medicine?	0.22	0.04
	Did you receive information on the side effects of the drug?	0.34	0.33
	What was the source of side effect information?	0.53	0.32
	What are the side effects you faced?	0.03	0.09
Gender			
	Do you know what osteonecrosis of the jaw is?	0.24	0.30
	Have you ever taken any of the bisphosphonate or any of the antiresorptive and antiangiogenic medications?	0.26	0.23
	What is the route of administration?	0.06	0.09
	When you first began taking the drug, were you transitioned from another drug?	0.40	0.31
	For which condition do you take the medicine?	0.78	0.81
	Did you receive information on the side effects of the drug?	0.76	0.83
	What was the source of side effect information?	0.35	0.34
	What are the side effects you faced?	0.29	0.43
Education			
	Do you know what osteonecrosis of the jaw is?	0.78	0.42
	Have you ever taken any of the bisphosphonates or any of the antiresorptive and antiangiogenic medications?	0.000	0.003
	What is the route of administration?	0.02	0.03
	When you first began taking the drug, were you transitioned from another drug?	0.65	0.57
	For which condition do you take medicine?	0.09	0.10
	Did you receive information on the side effects of the drug?	0.08	0.06
	What was the source of side effect information?	0.91	0.86
	What are the side effects you faced?	0.002	0.003

## Discussion

MRONJ is linked to significant morbidity, resulting in symptoms such as pain, tooth looseness, bad breath, tingling sensations, the formation of bone sequestra, and the development of fistulas either within the mouth or extending beyond it [21]. It was first documented by Teślak M et al. [22] in a study involving 36 patients receiving IV bisphosphonate treatment. These individuals experienced necrotic bone exposure within the oral cavity. Therefore, the present study was designed to assess the awareness levels of MRONJ among patients currently undergoing treatment with antiresorptive and antiangiogenic medications.

In the present study, a significant but small proportion of participants (34.5%) are aware of jaw osteonecrosis, with a statistically significant association (p=0.002). In similar studies, awareness reported among patients from Brazil was 9.2% [17], from Jordan 12.4% [18], and from Germany 32% [19], which was lower than our study, whereas the results were similar to a study conducted in Saudi Arabia (33.82%) [5]. Similarly, in a study by Migliorati CA et al. [20], only 28% could recall being informed about the potential side effects of bisphosphonate therapy. Our findings align with those of [23], concluding that 28.1% of individuals had an accurate understanding of MRONJ. However, this proportion is very small compared with other studies, such as the findings of [24]. In contrast, 94.6% of individuals in another study indicated they knew the term MRONJ [25]. The variance in awareness levels between studies could be attributed to several factors, including differences in the study populations, sampling methods, geographical locations, survey instruments employed, or the timing of data collection. Additionally, variations in the dissemination of information, education campaigns, or evolving healthcare practices over time could influence participants' awareness levels. Furthermore, the present study's findings indicate a need for more patient awareness regarding the potential side effects of bisphosphonate medications, as most respondents (30.7%) did not know the side effects. While physicians prescribing these drugs are expected to provide information, research suggests that healthcare professionals may need more knowledge about the drug's indications and possible adverse effects [26,27]. Consequently, patients' lack of awareness about the risks associated with these medications could contribute to MRONJ, especially since dentists may also be unprepared to assess this risk [27]. Therefore, conducting thorough and comprehensive patient histories becomes crucial to identify individuals within the drug user group who may not be aware of the potential risks involved. Meanwhile, when patients were questioned about the medical conditions for which bisphosphonates are used, a notable percentage accurately identified osteoporosis (41.8%) and cancer (13.5%) as common indications. These findings align with a study conducted by Hajmohammadi E et al. [28], involving 116 dentists, where 64.7% of participants correctly identified osteoporosis treatment. In the present study, there was a non-significant association between participants' age and awareness of MRONJ. The lack of a significant association between age and awareness of MRONJ may be attributed to several factors. Such MRONJ awareness campaigns and educational efforts may have been broadly distributed across age groups, as all are patients, and we assume patients of all age groups should have the knowledge, resulting in a relatively consistent level of awareness among participants of varying ages. Additionally, MRONJ may not necessarily be more prevalent or better recognized among older individuals, as it can occur in patients of different age groups who are taking medications that predispose them to this condition. Other variables such as educational level, access to healthcare information, or individual health concerns might have had a more prominent impact on awareness levels than age alone, making it difficult to establish a significant age-related association in this context.

Gender had a non-significant association with awareness of MRONJ in the present study. This may be due to MRONJ awareness campaigns and educational efforts that might have been designed and distributed in a way that reached both genders equally, resulting in a relatively balanced level of awareness among men and women. Additionally, MRONJ is a medical condition associated with specific medications, and its awareness may not be inherently tied to gender but rather to factors such as healthcare-seeking behavior, exposure to relevant healthcare information, and individual health concerns [29]. Men and women may have similar access to healthcare resources and information, leading to comparable levels of awareness. Furthermore, if MRONJ awareness campaigns were widespread and unbiased in their targeting, the influence of gender on awareness might have been minimized. Interestingly, in another study, of the sixty-eight patients, only 23 individuals, or 33.82%, were aware of jaw osteonecrosis, and the majority of those aware were female [5].

Meanwhile, education significantly affected the awareness of some questions, such as the use of bisphosphonate medications, routes, and side effects of the medication used. Our findings are in line with those of Keshwar S et al. [30]. Education plays a significant role in MRONJ awareness. It equips individuals with the knowledge, skills, and confidence needed to access, understand, and act upon healthcare information, ultimately leading to greater awareness and informed decision-making regarding their health and medication choices. It is indicated that postgraduate students had more awareness than undergraduate students [30].

While the study provides valuable insights into the awareness of MRONJ among individuals receiving antiresorptive and antiangiogenic medications, it is important to acknowledge certain limitations. The study's limitation was that it was conducted using a convenience sampling method, which may introduce bias. Participants included may not represent the broader population, potentially limiting the generalizability of the findings. The sample size of 110 participants might be relatively small, and the results may not fully capture the diversity of perceptions and awareness within the larger population; a multi-center approach could offer a broader perspective. Self-administered questionnaires may be prone to recall or social desirability bias. The questionnaire's limited scope might not capture all factors influencing awareness, such as health literacy or information sources. The cross-sectional design provides a snapshot, and longitudinal studies could reveal how awareness evolves over time. The study identifies a gap in awareness but does not delve into its causes; additional qualitative research could provide deeper insights. Participants aware of MRONJ might disproportionately participate, potentially leading to an overestimation of awareness levels. Addressing these limitations in future research would enhance the reliability and applicability of findings regarding MRONJ awareness among individuals on these medications.

## Conclusions

The findings underscore a notable gap in patient awareness, with only a minority of participants demonstrating knowledge of this potentially serious condition. While further research is needed to explore the specific factors contributing to this lack of awareness, our study highlights the importance of enhancing patient education and healthcare-provider communication regarding the risks associated with these medications. Improving awareness and early detection can play a pivotal role in minimizing the incidence and severity of MRONJ, ultimately leading to better oral health outcomes for patients receiving these treatments.
